# Study on the correlation between seven emotions and traditional Chinese medicine syndromes, serum IL-6 and IFN-γ in patients with primary lung cancer

**DOI:** 10.1016/j.clinsp.2026.100960

**Published:** 2026-04-15

**Authors:** Qun Wei, Chunmei Lu, Yurong Xu, Wei Xia

**Affiliations:** Shandong Public Health Clinical Center, Shandong University, Shandong 250013, China

**Keywords:** Seven emotions, Primary lung cancer, Traditional chinese medicine syndromes, IL-6, IFN-γ, Prospective Study

## Abstract

**Objective:**

To investigate the correlations among seven emotions, Traditional Chinese Medicine (TCM) syndromes, and serum levels of Interleukin-6 (IL-6) and Interferon-gamma (IFN-γ) in patients with primary lung cancer.

**Methods:**

In this prospective cohort study, 331 newly diagnosed, treatment-naïve lung cancer patients were enrolled. TCM syndrome differentiation was independently performed by two blinded senior physicians. Patients were categorized into four syndrome types: Lung Qi Stagnation with Phlegm and Blood Stasis (n = 95), Qi and Yin Deficiency (n = 127), Spleen Deficiency with Phlegm-Dampness (n = 36), and Yin Deficiency with Phlegm-Heat (n = 73). Emotional states were assessed using the “TCM Seven Emotions Scale”. Serum IL-6 and IFN-γ levels were measured via ELISA.

**Results:**

Specific emotions were predominantly associated with particular TCM syndromes (all p *<* 0.05). The Lung Stagnation with Phlegm and Blood Stasis group showed higher IL-6 levels, while the Qi and Yin Deficiency group had lower IFN-γ levels compared to other syndromes (p < 0.05). Crucially, Spearman's correlation analysis revealed a significant positive correlation between the total emotion score and serum IL-6 level (*rs* = 0.325, p *<* 0.001), and a significant negative correlation with IFN-γ level (*rs* = -0.391, p < 0.001).

**Conclusion:**

The seven emotions exhibit syndrome-specific expression patterns in lung cancer patients. Significant correlations were found between emotional states and the inflammatory markers IL-6 and IFN-γ, suggesting a potential integrative link between psychological state, TCM diagnosis, and systemic inflammation, which may inform clinical approaches.

## Highlights

• Seven emotions are linked to traditional Chinese medicine syndromes in lung cancer.
• Emotional states correlate with inflammation markers IL-6 and IFN-γ in patients.
• Findings suggest mind-body connections may inform future lung cancer care.

## Introduction

Primary lung cancer is the most common malignant tumor in China. From the pathological and therapeutic perspectives, lung cancer can be broadly classified into two major groups: Non-Small Cell Lung Cancer (NSCLC) and Small Cell Lung Cancer (SCLC). NSCLC accounts for about 80%‒85%, including histological subtypes such as adenocarcinoma and squamous carcinoma, while the remaining cases are SCLC.^1^ The pathogenesis of primary lung cancer is complex and diverse, involving smoking, occupational exposure, air pollution, genetic and molecular mechanisms, chronic inflammatory response, and lung cancer stem cells. These factors synergistically promote uncontrolled cell proliferation, genetic mutation, and oxidative stress, driving lung cancer initiation and progression.^2^ Among these, smoking has been recognized as the most important risk factor for lung cancer, accounting for 87% of cases.^3^ In Traditional Chinese Medicine (TCM), it is believed that the body's “righteous Qi” (vital energy) is crucial for maintaining life activities and defending against external pathogens. When righteous Qi is weakened, the body's resistance to disease decreases, making it more susceptible to external pathogens, which can lead to the development of lung cancer.^4^, ^5^, ^6^ In addition, emotional fluctuations such as prolonged mental stress, depression, and anger can harm the liver Qi, leading to impaired Qi circulation, which in turn affects lung function. This disruption in lung function may result in the onset of lung cancer.^7^^,^^8^ The seven emotions (joy, anger, worry, pensiveness, sadness, fear, and fright), as an important part of the theory of emotions in TCM, are the manifestations of the internal emotional activities of the human body, which are closely related to the functions of internal organs of the human body, and their imbalance may affect the Qi, blood, and body fluids, as well as organ function, and thus be related to the occurrence and development of tumors.^9^, ^10^, ^11^ In recent years, many studies have found a certain correlation between the seven emotions and TCM syndromes.^12^ such as the tendency of patients with depression to exhibit the liver qi stagnation syndrome and patients with anxiety to show heart-spleen deficiency syndrome. These studies provide a theoretical basis for exploring the relationship between the seven emotions and TCM syndromes in patients with primary lung cancer.

Serum Interleukin-6 (IL-6) and Interferon-Gamma (IFN-γ), as important inflammatory factors, are closely related to immune regulation and inflammatory response in tumors.^13^, ^14^, ^15^, ^16^, ^17^ According to relevant data, the expression of IL-6 and IFN-γ in lung cancer not only reflects the degree of inflammatory response^18^^,^^19^ but also relates to the immune microenvironment of the tumor. IL-6 promotes tumor cell growth by activating T-lymphocytes and inducing B-lymphocyte differentiation and immunoglobulin secretion, while IFN-γ plays a key role in activating cellular immunity and stimulating anti-tumor immune responses. Its anti-proliferative and pro-apoptotic functions make it a potential adjuvant immunotherapeutic agent. Currently, studies on IL-6 and IFN-γ in lung cancer have focused on their mechanisms of action in lung carcinogenesis, progression, and treatment, as well as their potential value as biomarkers. However, the relationship between the seven emotions and serum IL-6 and IFN-γ levels remains unclear. Therefore, this prospective study was designed to minimize recall bias and clarify the temporal sequence of assessments, aiming to investigate this correlation and provide a theoretical basis for TCM treatment of primary lung cancer.

## Data and methods

### Research subjects

This was a prospective, single-center cohort study. A total of 331 patients with newly diagnosed, treatment-naïve primary lung cancer were consecutively recruited from the Department of Oncology of the hospital between August 2022 and July 2024. All assessments, including TCM syndrome differentiation, seven emotions evaluation, and blood sampling, were conducted within one week after diagnosis and before the initiation of any surgical, chemotherapeutic, radiotherapeutic, or immunological anticancer treatment. This design ensured that the measured variables reflected the baseline state of the disease and were not confounded by treatment effects.

### Inclusion and exclusion criteria

Inclusion criteria: 1) All patients met the diagnostic and treatment standards for lung cancer[20] and were confirmed by pathological histology or cytology; 2) Patients had a well-functioning nervous system and were able to cooperate with examinations; 3) The Eastern Cooperative Oncology Group (ECOG) performance status score was 0‒2. Exclusion criteria: 1) Patients with an expected survival time of less than 6-months; 2) Patients with concurrent other malignant tumors or malignant diseases; 3) Patients with alcoholic fatty liver; 4) Patients with autoimmune diseases or hematological diseases; 5) Patients with severe cognitive impairment or communication barriers that prevented them from completing the questionnaire.

### TCM syndrome differentiation and distribution of the seven emotions

TCM Syndrome Differentiation: TCM syndrome differentiation was strictly performed according to the “Guiding Principles for Clinical Research of New Chinese Medicines” and relevant consensus. Two deputy chief physician-level TCM practitioners with over 10-years of clinical experience, who were blinded to the patients' emotional scale scores and laboratory results, independently conducted the assessments based on the patients' symptoms, tongue appearance, and pulse characteristics. Patients were classified into one of the following four syndrome types:

Lung Qi Stagnation with Phlegm and Blood Stasis Type: Characterized by symptoms such as unsmooth coughing, discomfort in expectorating phlegm, chest tightness, shortness of breath, chest, flank, and back pain, blood-streaked sputum, constipation, dark red tongue with yellow or white greasy coating, and a wiry pulse.

Qi and Yin Deficiency Type: Characterized by symptoms such as weak coughing with little sputum, low and faint cough sounds, blood-streaked sputum, fatigue, poor appetite, shortness of breath, dry mouth with little desire to drink, red tongue with a thin coating, and a weak or rapid thin pulse.

Spleen Deficiency with Phlegm-Dampness Type: Characterized by symptoms such as coughing with abundant sputum, chest tightness, poor appetite, fatigue, shortness of breath, abdominal distension, loose stools, pale and swollen tongue with teeth marks on the edges, white, greasy coating, and a soggy and slow pulse.

Yin Deficiency with Phlegm-Heat Type: Characterized by symptoms such as dry coughing with little sputum or dry cough without sputum, blood-streaked sputum, chest pain, shortness of breath, irritability, insomnia, dry mouth with constipation, tidal fever, night sweats, red tongue with little or thin yellow coating, and a rapid, thin pulse.

The emotional state of the patients was assessed using the “Traditional Chinese Medicine Seven Emotions Scale”,^20^^,^^21^ which has demonstrated good reliability and validity in Chinese populations with various chronic diseases.^22^^,^^23^ The use of this TCM-based emotional assessment is pertinent as the seven emotions are theorized to be intimately linked with visceral function and the pathogenesis of diseases, including cancer.^24^ While a formal validation study specifically in treatment-naïve lung cancer patients is lacking, the application of the scale provides preliminary evidence for its utility in this context, as reflected in the interpretable patterns of association found with TCM syndromes and biomarkers.

The scale evaluates the seven emotions (joy, anger, worry, pensiveness, sadness, fear, and fright) across 49-items. Each item is scored from 0 to 5, with total scores of 0 indicating no emotional distress, 1‒7 indicating mild distress, 8‒14 indicating moderate distress, and ≥ 21 indicating severe distress for each emotion domain. Although the seven emotions represent distinct constructs, the authors used a composite total score for exploratory analysis to provide an overall measure of emotional distress. This approach is justified for initial investigations of broad relationships, though the authors acknowledge it may mask differential effects of specific emotions. The total seven-emotion state score was the sum of all item scores. The questionnaire was self-administered by the patient in a quiet room, with a research assistant present to provide instructions if needed. Given the timing of assessment, the scale primarily captured the patients' current emotional state (state emotions) in response to their diagnosis and illness, rather than enduring personality traits (trait emotions).

### Serum IL-6 and IFN-γ detection

Fasting venous blood samples (4 mL) were drawn from the median cubital vein of all participants on the same day or the day after the TCM and emotional assessments. The samples were processed and stored at −80 °C until batch analysis. Serum levels of IL-6 and IFN-γ were measured using commercially available Enzyme-Linked Immunosorbent Assay (ELISA) kits (Sigma, Germany; Catalog numbers: RAB0306 and RAB0222, respectively) according to the manufacturer's instructions.

### Statistical analysis

SPSS 23.0 statistical software was used to analyze the data. The normality of the distribution for measurement data was assessed using the Shapiro-Wilk test. Since the data for the seven emotions score, IL-6, and IFN-γ were found to be non-normally distributed (p < 0.05 for all), these data are presented as medians. A priori power analysis was conducted using G*Power 3.1. To detect a medium effect size (*f* = 0.25) in ANOVA comparing four TCM syndrome groups with α = 0.05 and power = 0.80, a minimum sample size of 180 was required. The total sample size of 331 exceeded this requirement; however, subgroup analyses (especially for the Spleen Deficiency with Phlegm-Dampness group, n = 36) may be underpowered. The relationship between the seven emotion state scores and serum levels of IL-6 and IFN-γ was analyzed using Spearman's rank correlation analysis. For other continuous data that met normality assumptions, results were expressed as mean ± standard deviation (x̄±s). Comparisons between two groups were performed using the independent sample *t*-test, and comparisons among multiple groups were analyzed by one-way analysis of variance (ANOVA) or analysis of covariance (ANCOVA) where appropriate. Enumeration data were expressed as [n (%)] and compared using the χ^2^ test. A p-value of less than 0.05 (p < 0.05) was considered statistically significant.

## Results

### Baseline characteristics across TCM syndromes

The baseline clinical and demographic characteristics of the 331 patients, stratified by the four TCM syndrome types, are presented in Table 1. There were no statistically significant differences in the distribution of gender, age, KPS score, pathological type, or smoking index among the four syndrome groups (all p > 0.05). The effect sizes for these comparisons, as measured by Cramér's V, were all in the small range (V < 0.17), indicating that any observed distributional differences were not only statistically non-significant but also of negligible clinical magnitude. This suggests that the groups were well-balanced at baseline regarding these key characteristics.

**Table 1:** Baseline clinical and demographic characteristics of patients by TCM Syndrome type.

Characteristic	Total (n = 331)	Lung Stagnation with Phlegm and Blood Stasis (n = 95)	Qi and Yin Deficiency (n = 127)	Spleen Deficiency with Phlegm-Dampness (n = 36)	Yin Deficiency with Phlegm-Heat (n = 73)	p	Effect Size
Gender (%), n (%)						0.165	Cramér's V = 0.124
Female	156 (47.1)	41 (43.2)	54 (42.5)	20 (55.6)	41 (56.2)		
Male	175 (52.9)	54 (56.8)	73 (57.5)	16 (44.4)	32 (43.8)		
Age (years), n (%)						0.315	Cramér's V = 0.104
≤ 59	173 (52.3)	50 (52.6)	73 (57.5)	18 (50.0)	32 (43.8)		
> 59	158 (47.7)	45 (47.4)	54 (42.5)	18 (50.0)	41 (56.2)		
KPS score, n (%)						0.462	Cramér's V = 0.092
≤ 79	196 (59.2)	50 (52.6)	77 (60.6)	23 (63.9)	46 (63.0)		
> 79	135 (40.8)	45 (47.4)	50 (39.4)	13 (36.1)	27 (37.0)		
Pathological type (%), n (%)						0.277	Cramér's V = 0.108
Small cell lung cancer	60 (18.1)	18 (19.0)	19 (14.3)	5 (13.9)	18 (24.7)		
Non-small cell lung cancer	271 (81.9)	77 (81.0)	108 (85.0)	31 (86.1)	55 (75.3)		
Smoking index						0.300	Cramér's V = 0.099
< 400	164 (49.5)	45 (47.3)	59 (46.5)	23 (63.9)	37 (50.7)		
≥ 400	167 (50.5)	50 (52.6)	68 (53.5)	13 (36.1)	36 (49.3)		
TNM stage, n (%)						0.218	Cramér's V = 0.116
Ⅰ	42 (12.7)	12 (12.6)	15 (11.8)	5 (13.9)	10 (13.7)		
Ⅱ	65 (19.6)	18 (18.9)	22 (17.3)	7 (19.4)	18 (24.7)		
Ⅲ	138 (41.7)	40 (42.1)	53 (41.7)	15 (41.7)	30 (41.1)		
Ⅳ	86 (26.0)	25 (26.3)	37 (29.1)	9 (25.0)	15 (20.5)		
Comorbidities, n (%)						0.357	Cramér's V = 0.101
None	142 (42.9)	43 (45.3)	51 (40.2)	16 (44.4)	32 (43.8)		
Hypertension	98 (29.6)	26 (27.4)	38 (29.9)	9 (25.0)	25 (34.2)		
Diabetes mellitus	53 (16.0)	15 (15.8)	21 (16.5)	6 (16.7)	11 (15.1)		
Hypertension + Diabetes mellitus	38 (11.5)	11 (11.6)	17 (13.4)	5 (13.9)	5 (6.8)		

**Note:** TCM, Traditional Chinese Medicine; KPS, Karnofsky Performance Status.

*p-values were derived from Chi-Square tests. Effect sizes for categorical variables are reported as Cramér's V, where 0.06 ≤ V < 0.17 represents a small effect, 0.17 ≤ V < 0.29 a medium effect, and V ≥ 0.29 a large effect.

### Distribution of seven emotions across different TCM syndromes

After adjusting for age, gender, and TNM stage, there were significant differences in the prevalence of specific emotions among patients with different TCM syndromes, as determined by Chi-Square tests with FDR correction for multiple comparisons. The results were as follows: A significantly higher proportion of patients with “Lung Stagnation with Phlegm and Blood Stasis” syndrome exhibited “Anger”, “Worry”, and “Pensiveness” (FDR-corrected p-values were 0.004, < 0.001, and < 0.001, respectively). Patients with “Spleen Deficiency with Phlegm-Dampness” syndrome had a significantly higher prevalence of “Worry” and “Pensiveness” (both FDR-corrected p < 0.001). For patients with “Yin Deficiency with Phlegm-Heat” syndrome, “Fright” and “Fear” were significantly more common (both FDR-corrected p = 0.012). Patients with “Qi and Yin Deficiency” syndrome showed a significantly higher proportion of “Sadness” and “Fear” (FDR-corrected p-values were 0.024 and 0.012, respectively). The prevalence of “Joy” did not differ significantly among the syndrome groups (FDR-corrected p = 0.784). The total seven emotions state score, however, was significantly different across syndromes (p < 0.001), with the Spleen Deficiency with Phlegm-Dampness and Yin Deficiency with Phlegm-Heat groups exhibiting significantly lower median total seven emotions scores than the Lung Stagnation with Phlegm and Blood Stasis and Qi and Yin Deficiency groups. Detailed data are presented in Table 2.

**Table 2:** Distribution of seven emotions and total score across different TCM syndromes.

Group	Joy	Angry	Worry	Pensiveness	Sadness	Fear	Fright	Total Score
Lung Stagnation with Phlegm and Blood Stasis (n = 95)	2 (2.1)	56 (58.9)	63 (66.3)	71 (74.7)	45 (47.4)	47 (49.5)	50 (52.6)	175.0 (142.0, 225.5)
Qi and Yin Deficiency (n = 127)	7 (5.5)	43 (33.9)	52 (40.9)	47 (37.0)	84 (66.1)	80 (63.0)	58 (45.7)	175.0 (145.0, 205.0)
Spleen Deficiency with Phlegm-Dampness (n = 36)	1 (2.8)	12 (33.3)	22 (61.1)	30(83.3)	15(41.7)	14 (38.9)	12 (33.3)	125.0 (108.5, 147.5)
Yin Deficiency with Phlegm-Heat (n = 73)	2 (2.7)	30 (41.1)	28 (38.4)	32 (43.8)	35 (47.9)	48 (65.8)	49 (67.1)	112.0 (89.5, 142.0)
**FDR-corrected *P***	0.784	<0.001*	<0.001*	<0.001*	0.011*	0.002*	<0.001*	<0.001*

Note: TCM, Traditional Chinese Medicine. Data for individual emotions are presented as n (%). The total score is presented as median (interquartile range). FDR-corrected p < 0.05 indicates significant differences across groups, and p < 0.001 applies to the total score and the marked emotional domains.

### Differences in serum IL-6 and IFN-γ levels among patients with different TCM syndromes

After adjusting for age, gender, and TNM stage using ANCOVA, there were significant differences in serum IL-6 and IFN-γ levels among patients with different TCM syndromes (*F* values were 64.21 and 88.75, respectively, and uncorrected p-values were both < 0.001). After further correction using the Benjamini-Hochberg False Discovery Rate (FDR) procedure, the differences remained statistically significant (FDR-corrected p-values were both < 0.001). See Table 3.

**Table 3:** Comparison of serum IL-6 and IFN-γ levels across different TCM syndromes.

Group	IL-6 (pg/mL)	IFN-γ (pg/mL)
Lung Stagnation with Phlegm and Blood Stasis (n = 95)	18.8 (16.2, 21.4)	2.9 (2.3, 3.6)
Qi and Yin Deficiency (n = 127)	17.1 (14.9, 21.2)	2.7 (2.1, 3.3)
Spleen Deficiency with Phlegm-Dampness (n = 36)	12.8 (11.6, 14.9)	4.7 (3.8, 5.8)
Yin Deficiency with Phlegm-Heat (n = 73)	11.2 (9.6, 14.1)	4.8 (3.8, 6.5)
**p-value (FDR-corrected)**	<0.001*	<0.001*

Note: TCM, Traditional Chinese Medicine; IL-6, Interleukin 6; IFN-γ, Interferon-gamma. Data are presented as median (interquartile range). FDR-corrected p < 0.001 indicates significant differences across groups.

### Relationship between the seven emotional state scores and serum levels of IL-6 and IFN-γ

**Fig. 1:**
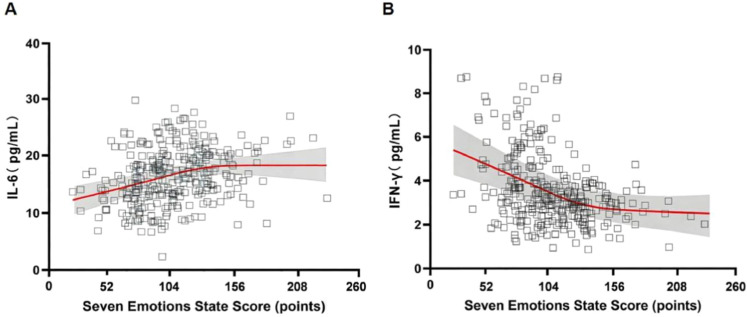
Correlation between seven emotions state score and serum IL-6 and IFN-γ levels. (A) Correlation between seven emotions state score and serum IL-6 level (pg/mL); (B) Correlation between seven emotions state score and serum IFN-γ level (pg/mL). IL-6, Interleukin 6; IFN-γ, Interferon-gamma; *rs*, Spearman's Rank correlation coefficient. Grey shaded area represents the confidence interval of the fitted curve.

Spearman's rank correlation analysis revealed a significant positive monotonic relationship between the total seven emotions state score and serum IL-6 level (*rs* = 0.325, p < 0.001), and a significant negative monotonic relationship with serum IFN-γ level (*rs* = −0.391, p < 0.001). See Table 4 and Fig. 1.

**Table 4:** Relationship between seven emotions state score and serum IL-6 and IFN-γ levels.

Index	Spearman's Correlation	Multiple Linear Regression	Standardized β (95% CI)	p_adj
	*rs*	p		
IL-6	0.325	<0.001	0.28 (0.15 to 0.41)	<0.001
IFN-γ	−0.391	<0.001	−0.35 (−0.49 to −0.21)	<0.001

Note: IL-6, Interleukin 6; IFN-γ, Interferon-gamma; *rs*, Spearman's Rank correlation coefficient; β, Standardized regression coefficient; CI, Confidence Interval.

## Discussion

In this prospective study of treatment-naive primary lung cancer patients, the authors found specific associations between TCM syndrome types, emotional states, and serum inflammatory markers. The correlational design identifies relationships but cannot determine causality. The observed patterns likely reflect complex interactions between psychological, traditional medical, and immunological factors in lung cancer.

Consistent with TCM theory, specific emotions were linked to different TCM syndromes. Anger, worry, and pensiveness were associated with the Lung Qi Stagnation with Phlegm and Blood Stasis pattern. In TCM, the liver regulates Qi flow, and anger is its predominant emotion. Constrained liver Qi may impair lung function, potentially contributing to phlegm and blood stasis formation.^25^^,^^26^ Worry and pensiveness, linked to the lungs and spleen respectively, may further disrupt Qi movement.

For the Qi and Yin Deficiency pattern, sadness and fear were prominent. Sadness dissipates Lung Qi, while fear weakens the Kidneys.^5^^,^^27^ In patients with Qi and Yin deficiency, these emotions may both reflect and exacerbate the depletion of vital energy.

The authors found differential levels of serum IL-6 and IFN-γ across TCM syndromes. The Lung Qi Stagnation with Phlegm and Blood Stasis group had higher IL-6 levels (median: 18.8 pg/mL). These levels are consistent with the elevated IL-6 reported in lung cancer patients (typically 10‒30 pg/mL)^28^ and align with the pro-inflammatory state characteristic of blood stasis in TCM theory.^29^^,^^30^ Qi and blood stagnation may create a microenvironment conducive to inflammation.^31^

The Qi and Yin Deficiency group showed lower IFN-γ levels (median: 2.7 pg/mL). While IFN-γ generally promotes anti-tumor immunity, its role in cancer is complex. Low IFN-γ could indicate immune suppression (“Wei Qi” insufficiency in TCM) but might also reflect tumor-induced T-cell exhaustion or other immune escape mechanisms.^32^ The interpretation requires caution as both immune dysfunction and adaptive responses could contribute to low IFN-γ levels.

The observed associations between emotional distress and inflammatory markers (IL-6 and IFN-γ) suggest links between psychological states and systemic inflammation. From a psychoneuroimmunology perspective, chronic emotional distress activates neuroendocrine pathways, including the sympathetic-adrenomedullary axis and hypothalamic-pituitary-adrenal axis.^33^ This activation can stimulate pro-inflammatory cytokine production like IL-6 while potentially suppressing IFN-γ-mediated cellular immunity.^34^ The cross-sectional design cannot determine whether emotions influence inflammation, inflammation affects emotions, or both result from other factors like disease severity.

The present study has several limitations. The cross-sectional design precludes causal conclusions. Although the authors adjusted for age, gender, and TNM stage, unmeasured confounders may influence the observed relationships. The absence of healthy or other cancer controls limits the specificity assessment. The Spleen Deficiency with Phlegm-Dampness subgroup comprised 36 patients, which, while adequate for inclusion in group-level comparisons, limits the power for independent subgroup analyses. Consequently, the authors did not perform subgroup-specific correlation analyses between individual emotions and biomarkers for this group, and findings pertaining specifically to this syndrome type should be considered preliminary and require validation in larger cohorts. While the authors used a composite emotion score for initial exploration, future research should examine specific emotions' unique relationships with biomarkers. The modest sample size in some subgroups may limit statistical power for detailed comparisons.

In summary, the authors document associations between TCM syndromes, emotional states, and inflammatory markers in treatment-naive lung cancer patients. These findings highlight the interplay between psychological, traditional medical, and immunological factors. The present results do not imply causation but highlight directions for future research. Prospective longitudinal studies are needed to clarify temporal relationships. Research with control groups and comprehensive confounding factor assessment will help validate these findings. Experimental and interventional studies could determine whether modulating emotional patterns influences inflammation. Integrating psychological care with TCM syndrome differentiation may offer novel adjunctive strategies for improving patient well-being and outcomes.

## Ethics

The present study was approved by Shandong Public Health Clinical Center, Shandong University (Approval n° GWLCZXEC2022–38). This study was conducted according to the principles of the Declaration of Helsinki. The participants are aware of and consent to the publication of this study in accordance with the established procedures.

## Data availability

The datasets generated and/or analyzed during the current study are available from the corresponding author upon reasonable request.

## Conflicts of interest

The authors declare no conflicts of interest.
